# INSPIRE: a healthcare-specialized instructional design methodology for high-stakes e-learning in the era of artificial intelligence

**DOI:** 10.1186/s12909-026-10173-2

**Published:** 2026-08-18

**Authors:** Hilalah Alturkistani, Fahad Almalki

**Affiliations:** Saudi Commission for Health Specialties, Riyadh, Kingdom of Saudi Arabia

**Keywords:** Instructional design, Healthcare e-learning, Continuing professional development, Artificial intelligence, Medical education, Quality assurance, INSPIRE methodology

## Abstract

**Background:**

Healthcare e-learning operates under pressures that are difficult to satisfy simultaneously: it must be evidence-based, regulator-acceptable, clinically credible, and delivered on time. Established instructional design models, including ADDIE, the Successive Approximation Model, Dick and Carey, and Kemp, were not developed with these combined demands in mind and predate the routine use of artificial intelligence (AI) in content production. The field lacks a methodology that combines the structured governance required for regulated healthcare training, particularly in postgraduate medical education and continuing professional development (CPD), with the responsiveness needed to keep pace with evolving clinical evidence and AI-enabled production.

**Methods:**

This article develops and presents INSPIRE, a healthcare-specialized instructional design methodology, as a conceptual contribution. The methodology was developed through a structured synthesis of the strengths and limitations of established instructional design models, integration of recent evidence on artificial intelligence in medical education, and iterative practical refinement in a national health professions education setting. The framework is described in relation to its theoretical foundations, systematically mapped against four established alternatives on six dimensions relevant to healthcare, and illustrated through phase-level integration of AI-supported tasks paired with explicit human checkpoints.

**Results:**

INSPIRE is a seven-phase agile-phased hybrid framework comprising Intake, Needs Analysis, Structure, Production, Implementation, Review, and End Closure. It treats early technical integration, continuous quality assurance, and human-in-the-loop use of artificial intelligence as core design commitments rather than optional additions. The framework addresses three combined gaps that no single existing model resolves: it integrates and organizes agility and governance into a single workflow suited to compliance-bound healthcare education; it formalizes the role of artificial intelligence across the lifecycle while retaining clinical and pedagogical accountability with human experts; and it makes quality review a continuous rather than terminal function.

**Conclusions:**

INSPIRE integrates phase-based governance, iterative production, continuous quality assurance, and human-in-the-loop AI use in one workflow built for healthcare e-learning. The contribution is conceptual; empirical validation against time-to-delivery, learner outcomes, and accreditation-quality measures is the next step. National regulators, postgraduate training boards, and CPD providers are the intended adopters.

## Introduction

Digital learning has moved from a backup channel for clinical education to an integral part of how the workforce is built. In this article, e-learning refers to any structured educational activity delivered through digital platforms, including asynchronous modules, virtual patient scenarios, simulation-based cases, and blended online-offline training designed for health professionals. The primary application domain for INSPIRE is postgraduate medical education and continuing professional development (CPD), where accreditation, clinical evidence-currency, and multi-stakeholder governance impose the greatest combined pressures on design. Trainees encounter their first immersive scenarios online. Clinicians complete the bulk of their CPD through digital platforms. National regulators increasingly accept and require digital delivery as a legitimate route to credentialing and re-credentialing [1]. In that context, the question is not whether e-learning matters in healthcare; the question is whether the way we design it is fit for the demands now placed on it.

Those demands are unusual. A course on diabetes management may need to reflect a guideline that changed last quarter. A sepsis recognition simulation must withstand clinical scrutiny while passing an accessibility audit. A national CPD module may need to launch within a fixed window, meet national regulatory standards, and withstand multi-stakeholder review by clinicians, instructional designers, and platform engineers. The pressure is rarely on one dimension at a time. Designers are asked to be fast, rigorous, compliant, and clinically credible together [2, 3].

Instructional design models that the field still relies on were not built around that combination. The Analysis, Design, Development, Implementation, Evaluation (ADDIE) framework gives a clear, systematic structure, but proceeds in a way that makes late discoveries costly [4].The Successive Approximation Model (SAM) gains agility through rapid prototyping but is harder to govern in regulated settings [5]. Models from the Dick and Carey and Kemp traditions are pedagogically rich but predate the way artificial intelligence (AI) now reshapes content production end-to-end [4]. Each gives part of what healthcare e-learning needs. None gives the whole.

This article introduces INSPIRE, a methodology developed in response to that gap, and frames it as an original contribution to the literature on instructional design for healthcare. INSPIRE (Intake, Needs Analysis, Structure, Production, Implementation, Review, End Closure) is an agile-phased hybrid: predictable enough to support governance, flexible enough to absorb the iterative work that complex clinical content actually requires. It treats early technical integration, continuous quality assurance, and human-in-the-loop AI use as foundational commitments rather than as optional extras.

The argument of this article is threefold. First, healthcare e-learning has reached a point where the traditional choice between structured and agile design is no longer adequate, and a hybrid is needed. Second, AI is now embedded in the production work of instructional design teams to a degree that requires it to be modelled in the framework itself rather than treated as a tool used outside the workflow. Third, in regulated, high-stakes domains such as postgraduate medical education and CPD, quality and compliance must be designed in, not inspected in at the end. INSPIRE is presented here as one way to do all three within a single, usable framework.

The remainder of the article is organized as follows. Section  2 reviews related work in instructional design and AI-enabled education. Section  3 sets out the conceptual and operational gap that INSPIRE addresses. Section  4 introduces the framework, its design principles, and its seven phases. Section  5 compares INSPIRE with established models. Section  6 details AI integration across the lifecycle. Section  7 discusses theoretical and practical implications. Section  8 concludes.

## Background and related work

Instructional design as a field has long oscillated between two impulses: the wish for systematic predictability and the wish for responsiveness. ADDIE represents the first impulse. Its five-stage logic of analysis, design, development, implementation, and evaluation gave the field a shared vocabulary and a defensible workflow [4]. The cost of that clarity is well documented. ADDIE pushes evaluation to the end, which is the worst possible time to discover a structural problem. Its handoffs between stages can entrench silos between instructional designers, subject matter experts (SMEs), and technical teams. In domains where requirements move during development, those costs accumulate.

SAM, developed by Allen, was a deliberate response [6]. Instead of moving once through a long line of stages, SAM moves repeatedly through short cycles of prototyping, review, and refinement. Comparative studies suggest learners and student-teachers often prefer SAM in dynamic settings because change is built into the rhythm of the work [5]. The trade-off runs in the opposite direction from ADDIE: SAM tolerates fluid documentation and informal governance in a way that can be uncomfortable when external auditors will eventually arrive.

Other models extend the field in useful directions. Dick and Carey emphasize systemic alignment among objectives, assessment, and instruction. Kemp’s nine-element model centers the learner and resists strict sequence. Both contribute conceptual depth, but both predate today’s working conditions: distributed teams, AI-assisted production, and learning analytics layered on top of national compliance regimes [4].

### Agile influences and appetite for hybrid models

Outside of instructional design, agile methods have reshaped how complex digital products are built for more than two decades. Their core ideas, which are short cycles, continuous integration, early feedback, and cross-functional teams, have found their way into educational design through SAM and through practitioner adaptations of agile to course development. What is increasingly being asked for, in scholarly and practitioner writing alike, is not pure agility but a hybrid that retains the documentation, traceability, and audit-friendliness of structured models while picking up the responsiveness of agile work [4, 5]. Healthcare e-learning is one of the clearest cases where this hybrid is needed, because the same teams must simultaneously satisfy clinicians, regulators, and learners.

### Artificial intelligence in clinical education

AI is no longer experimental in healthcare. Alowais describes its rapidly widening footprint in clinical decision support, diagnostic imaging, workflow optimization, and patient-facing communication [2]. That clinical penetration matters for education in two ways. It changes what clinicians need to learn, where competence with AI tools is itself becoming a CPD requirement, and it changes what is possible in the production of educational content.

Recent reviews of AI in medical education have moved from speculation to mapping. Gordon and colleagues [3], in BEME Guide No. 84, synthesized 278 publications and found AI applications spanning admissions, teaching, assessment, and clinical reasoning, with most evidence still concentrated in undergraduate settings in North America and Europe. They flag postgraduate medical education and CPD as comparatively under-studied, which is precisely where INSPIRE is positioned.

Barra and colleagues provide one of the more concrete demonstrations of AI’s potential in healthcare-specific instructional design [7]. Their agentic AI workflow for simulation scenario design uses prompt chaining, decomposition, parallelization, and retrieval-augmented generation across specialized AI agents. They report development-time reductions of approximately 70–80% while remaining aligned with the INACSL and ASPiH simulation standards. The authors also stress what AI does not remove: the need for clinical review, the awareness of bias in model outputs, and the requirement for human oversight. That balance which speed gained, accountability preserved, is one of the central design commitments of INSPIRE.

### Healthcare and the regulatory body

Healthcare e-learning is not a generic category. It is shaped by the regulatory body that accepts the credit, the standards organization that audits the design, and the institutional context in which clinicians actually train. McMahon and colleagues [1] describe how the Saudi Commission for Health Specialties (SCFHS) overhauled its CPD ecosystem to align with international standards, growing accredited CPD activities from roughly 1,000 in 2002 to 10,500 in 2022. That kind of scaling is only possible if the underlying design processes are also reformed; you cannot run ten thousand activities a year through a workflow built for one thousand. The Saudi case is not unique. Comparable scaling pressures exist in jurisdictions ranging from the United Kingdom’s General Medical Council framework to the Accreditation Council for Continuing Medical Education’s (ACCME) system in the United States. The methodological question is the same in each case: what design approach allows quality, volume, and pace to coexist?

## The research gap

The literature reviewed above points to a three-layer gap, and INSPIRE is constructed against all three.

i. *The first layer is operational.* Established models split the field into structured options that struggle to flex (ADDIE, Dick and Carey) and agile options that struggle to govern (SAM, Kemp). Healthcare e-learning needs both qualities at once: documented enough to survive audit, flexible enough to absorb late clinical feedback, protocol changes, and evolving practice standards [4, 5].
ii. *The second layer is technological.* Most established instructional design models were written before generative AI became part of the production stack. AI is now used to draft objectives, propose scenarios, generate narration, scaffold assessment items, and run first-pass quality checks [3, 7]. When AI is bolted onto a model that does not anticipate it, it generates inconsistent practice and uneven quality. A framework that names the AI-supported tasks alongside their human checkpoints is a different proposition from one that treats AI as a tool used somewhere outside the diagram.
iii. *The third layer is contextual.* Healthcare education has compliance, evidence-currency, and learner-stake requirements that general corporate or academic e-learning does not share to the same degree [1, 2]. A clinician who emerges from a CPD module with limited knowledge is not merely an unhappy customer; the downstream risk extends to patients. Frameworks built for generic digital learning underestimate this, and frameworks built before the regulatory landscape matured do not address it at all.

Overall, these three layers describe a methodological space that is not adequately filled by any single existing model. INSPIRE was developed as one occupant of that space.

## The INSPIRE Framework

### Conceptual positioning

INSPIRE is best understood as an agile-phased hybrid. It keeps the visible architecture of phase-based design because that architecture is what regulators, governance committees, and clinical leaders can read at a glance. Inside each phase, however, the methodology assumes the iterative behavior that complex clinical content actually requires: short loops, parallel work, fast feedback, and willingness to revise. The hybrid is not a compromise between ADDIE and SAM; it is a deliberate combination of phase-visibility for governance and cycle-behavior for delivery.

**Fig. 1:**
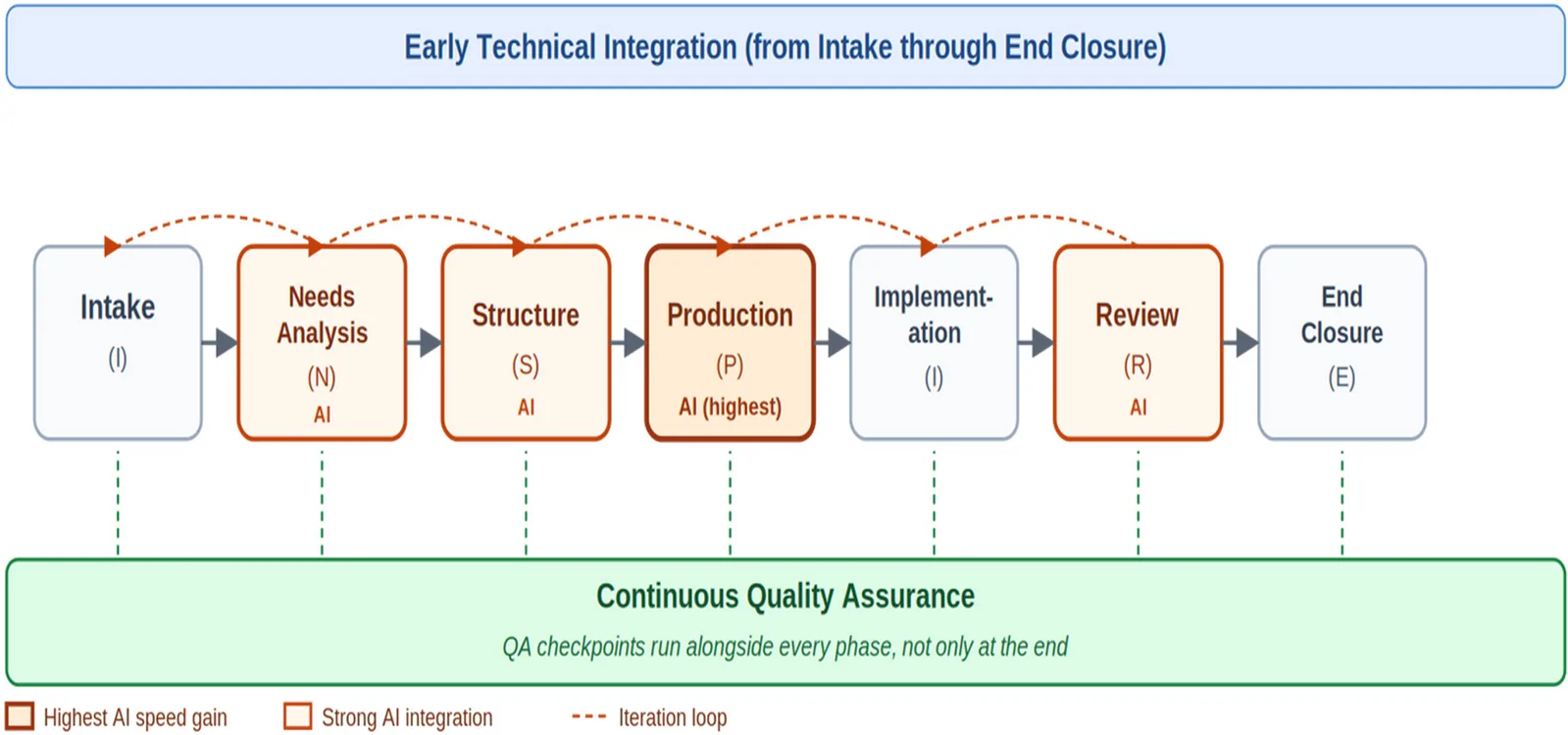
The INSPIRE methodology. Seven sequential phases (Intake, Needs Analysis, Structure, Production, Implementation, Review, End Closure) provide governance-visible architecture; dashed iteration arrows show the agile behavior available between adjacent phases. Two cross-cutting bands, Early Technical Integration above and Continuous Quality Assurance below, operate throughout the lifecycle. Needs Analysis, Structure, Production, and Review are highlighted as phases of strong AI integration, with Production carrying the greatest AI-assisted speed gain and consequently requiring the strictest subject-matter-expert sign-off. Dashed green lines indicate that Continuous Quality Assurance operates as feedback loops running from every phase, not only as a terminal check

Three design commitments distinguish INSPIRE from its predecessors. First, technical participation begins at intake rather than appearing at production. Second, quality assurance is treated as a continuous function with a dedicated consolidation phase, not as a terminal inspection. Third, AI is named explicitly in the workflow, mapped to specific tasks, and paired with explicit human-in-the-loop checkpoints. Together, these commitments shape every phase that follows. Figure 1 summarizes the framework.

### The seven phases

#### Intake (I)

Intake is where a request becomes a project. The work of this phase is to clarify the educational need, identify stakeholders, surface constraints, and produce a brief project charter that establishes scope and approvals. In healthcare, the request often arrives with implicit urgency: a guideline has changed, a quality indicator is failing, a cohort of trainees needs to develop a competency the curriculum did not previously assess. Treating intake as a thinking phase rather than a paperwork phase reduces the rework that otherwise propagates through the project.

Two practices distinguish INSPIRE’s intake from its equivalent in ADDIE. The Technical Lead is named on the charter from day one. And the project charter records not only what will be built but what will be measured at closure, so that evaluation criteria do not appear retrospectively.

#### Needs analysis (N)

Needs analysis examines the learner population, the clinical context, the gap to be closed, and the outcomes the activity must produce. Audience analysis goes beyond demographics to include role-based competencies, evidence sensitivity, and the practice settings learners actually work in. Learning objectives are aligned to recognized competency frameworks where they exist, such as the CanMEDS roles or local equivalents [8, 9].

AI enters needs analysis through gap mapping, learner profiling, and first-draft objective generation. These are first-pass artifacts; the SME retains authority over relevance and accuracy. The point of using AI here is not to reach the answer faster but to reach the conversation faster, giving SMEs and instructional designers a concrete starting point about learner needs, competency alignment, and objective phrasing that is easier to critique and refine than to author from scratch.

#### Structure (S)

Structure converts analyzed needs into an actionable blueprint: module outlines, storyboards, prototypes, instructional patterns, and technical specifications. This is where conventional workflows tend to bottleneck because review cycles among multiple stakeholders are slow when conducted in series. INSPIRE addresses this by running them in parallel, supported by shared artifacts that all roles can interact with at the same time.

For simulation-rich or case-based content, AI-supported drafting of branching scenarios accelerates the structure phase considerably [7]. The output is reviewed against simulation standards before any production effort begins.

#### Production (P)

Production turns the blueprint into actual learning assets, multimedia, interactions, assessments, narration, and the assembly of clinical scenarios. It is the most resource-intensive phase in any e-learning project, and consequently the phase where the speed gains from AI are most visible and most consequential. AI-supported drafting of clinical dialogues, image generation for non-photoreal medical illustration, and voice-over support all sit in this phase.

The risk in this phase is not speed; it is the acceptance of speed as a substitute for quality, treating polished AI output as if it had already been reviewed. INSPIRE treats clinical SME sign-off on AI-generated content as a non-optional checkpoint, regardless of how confident the model output looks. Barra and colleagues make the same point in the context of simulation: agentic workflows can compress development time by 70–80%, but the saved time must, in part, be reinvested in review rather than fully banked as efficiency [7].

#### Implementation (I)

Implementation deploys the activity into the learning environment. Activities include packaging in interoperable formats such as SCORM or xAPI, integration with the learning management system, competency-tracking configuration, learner communications, and pilot testing. In healthcare contexts, this phase often also involves coordination with the national accreditation pathway and with any required reporting to the regulator.

Pilot testing is not a courtesy in INSPIRE; it is part of the workflow. A small representative cohort runs the activity before full release, and pilot data feeds back into a final round of refinement. This is how INSPIRE earns its quality claim: by closing the loop before learners encounter the full version, not after.

#### Review (R)

Review is the phase that most clearly distinguishes INSPIRE from ADDIE. In ADDIE, evaluation lives at the end of the line. In INSPIRE, review is a continuous function that runs alongside every phase, with a dedicated phase for consolidation. Quality assurance checks happen at intake, at structure sign-off, at production milestones, and at deployment, not only at the end.

The consolidated review phase integrates four streams: standards-based audit against external rubrics such as Quality Matters [10] and national regulatory criteria; learner feedback synthesis; accessibility evaluation; and performance and learning analytics. AI assists with feedback clustering and pattern detection across large volumes of responses. Final interpretation stays with the design team.

#### End closure (E)

Closure formalizes the project’s exit. The deliverables are a final report, an archived version of all project files, a documented set of lessons learned, and a formal sign-off from stakeholders. Closure tends to be the phase that is skipped in practice when teams move directly to the next project. INSPIRE treats it as the moment where individual projects are converted into organizational knowledge: which storyboards worked, which AI prompts produced usable first drafts, which review steps caught the most defects, which integrations broke under load.

### Cross-cutting features

Three features run across the phases rather than belonging to any one of them.

i. Early technical integration. A Technical Lead, an Instructional Technologist, and a Multimedia Developer are present from intake. The intent is to surface platform constraints, integration requirements, and accessibility considerations before they become rework. In conventional models, these roles arrive at production, by which point structural assumptions are already cemented.
ii. Human-in-the-loop AI use. AI assists every phase of the workflow, including End Closure, where it can accelerate drafting of the final report, synthesis of lessons-learned notes, and review of prompt libraries for reuse, but it does not own any decision. The role of AI in INSPIRE is to draft, propose, cluster, and accelerate; the role of humans is to evaluate, accept, revise, and accept accountability. This is the same human–AI division that Alowais and colleagues describe as essential for safe clinical AI adoption, applied here to instructional design [2].
iii. Continuous quality assurance. Quality criteria are written into the charter at intake and revisited at each phase boundary. This is what allows INSPIRE to claim that quality is designed in rather than inspected in. The downstream effect is that the dedicated Review phase becomes consolidation work, not the first moment of evaluation.

## Comparative analysis with established models

**Fig. 2:**
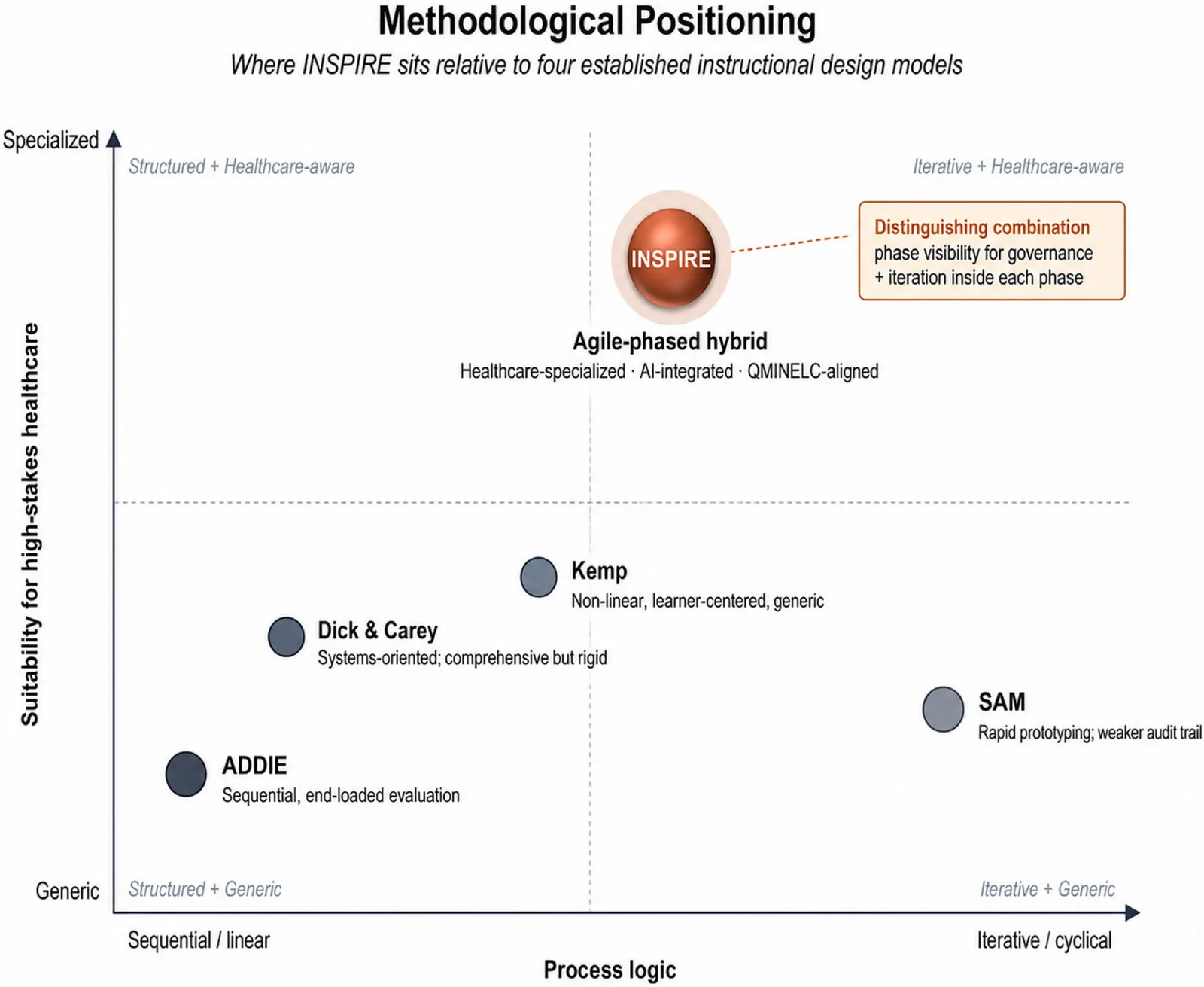
Methodological positioning. ADDIE clusters in the structured–generic quadrant; SAM in the iterative–generic quadrant; Dick and Carey and Kemp in between, with neither explicitly built for healthcare. INSPIRE occupies a distinct position by combining phase visibility for governance with iterative behavior inside each phase, and by being purpose-built for high-stakes clinical learning

The vertical placement of INSPIRE on the healthcare-suitability axis in Fig. 2 reflects three characteristics that distinguish healthcare e-learning from generic digital learning: the evidence base moves faster than fixed curricula can track; the audit burden imposed by accreditation and regulatory bodies is higher than in most settings; and the downstream consequence of weak learning is patient harm rather than commercial inconvenience. INSPIRE was purpose-built around these conditions, whereas the other four frameworks were built for broader educational contexts and adapted for healthcare where used. The rationale for INSPIRE’s position on this axis is therefore based on design intent rather than empirical benchmarking; readers whose primary experience is outside high-stakes clinical settings may reasonably position it lower on the suitability axis, and empirical validation is set out as a research agenda in Sect.  8.

Figure 2 plots INSPIRE alongside four established instructional design models on two dimensions that matter most for high-stakes healthcare contexts: process logic (sequential versus iterative) and suitability for healthcare-specific demands (generic versus specialized). The positioning is subjective to the authors and reflects the dominant emphasis of each model as characterized in the literature; empirical comparison is set out as a research agenda in Sect.  8.

Table 1 expands the same comparison along six dimensions relevant to healthcare e-learning.

**Table 1:** Comparative analysis of INSPIRE and four established instructional design models

Dimension	INSPIRE	ADDIE	SAM	Dick & Carey / Kemp
Process logic	Agile-phased hybrid; seven phases run in linear or iterative mode depending on project type.	Linear and sequential; each phase completes before the next begins.	Iterative and cyclical; prototyping, review, and refinement repeat in short loops.	Systems-oriented (Dick & Carey) or learner-centered non-linear (Kemp); both prescriptive in scope.
Flexibility	Combines a stable phase architecture with iterative behavior inside phases.	Low; changes after a phase is closed are costly.	High; designed for change.	Moderate; flexibility depends on team practice rather than the model.
Technical integration	From Intake onward; Technical Lead, Instructional Technologist, and Multimedia Developer named on charter.	Typically at the Development stage; technical realities surface late.	Iterative team includes technical roles but timing varies.	Not explicitly modelled; depends on team configuration.
Quality assurance	Continuous, with a dedicated consolidation phase; aligned to external standards (e.g., Quality Matters [10]).	End-loaded; evaluation is the final phase.	Formative through iteration; less formal documentation.	Systematic alignment focus; no embedded continuous QA loop.
AI integration	Foundational; AI is mapped to specific phases with explicit human-in-the-loop checkpoints.	Not anticipated by the model; usually retrofitted.	Possible within iterations; not foundational.	Not anticipated; predates current AI tooling.
Healthcare and CPD fit	Designed for high-stakes regulated environments; balances agility with compliance.	Strong on documentation, weak on agility; struggles with evidence-pace.	Strong on responsiveness, weaker on auditability.	Conceptually strong, operationally less prescriptive for compliance settings.

The pattern in Table 1 is not that INSPIRE is uniformly superior. ADDIE remains a stronger teaching framework for novice instructional designers. SAM remains stronger when the deliverable is exploratory, and the audit burden is light. The argument is more specific: in regulated, AI-enabled, fast-moving healthcare and CPD settings, no other framework holds all of structure, agility, technical integration, continuous QA, and explicit AI mapping in one place. That combination is what INSPIRE is built to provide.

## AI integration across the INSPIRE lifecycle

**Fig. 3:**
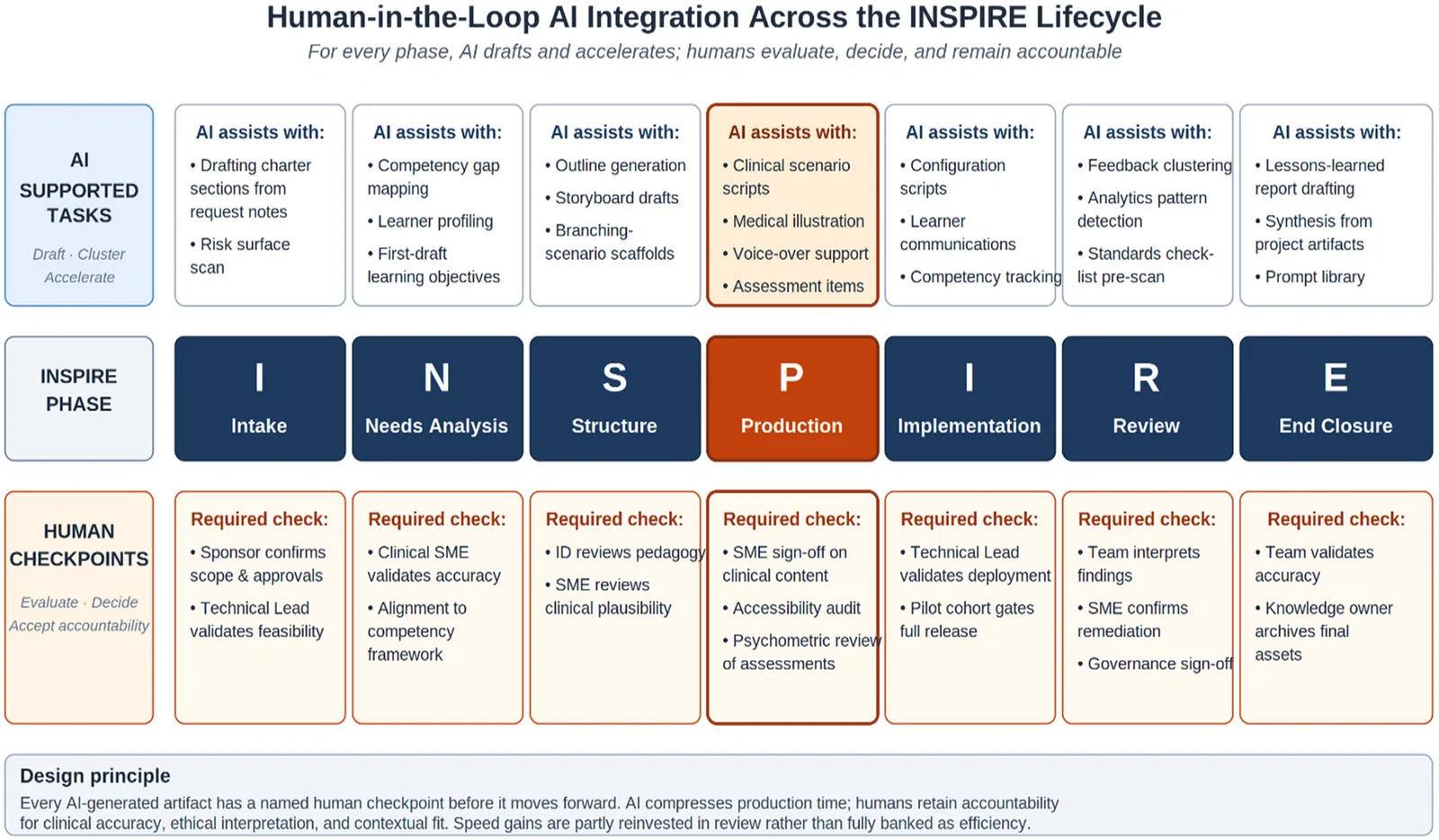
Human-in-the-loop AI integration across the INSPIRE lifecycle. Each column represents one phase. The top cell lists AI-supported tasks; the bottom cell lists the human checkpoint required before any AI-generated artifact moves forward. The Production phase is highlighted because it carries both the largest speed gain and the largest review burden

Figure 3 shows the human-in-the-loop pattern that runs through every INSPIRE phase. AI sits in the top lane, performing draft, cluster, and acceleration work. Phases sit in the middle. Human checkpoints sit in the bottom lane, performing evaluate, decide, and accountability work. The pattern is one of redistribution rather than replacement: automation does what it does well, and human time is reinvested in what only humans can do, judgment about relevance, clinical accuracy, ethical interpretation, and contextual fit [2, 3, 7].

Table 2 lays the same information out in tabular form, pairing each AI-supported task with the human checkpoint that must clear it.

**Table 2:** AI-supported tasks and human checkpoints across the INSPIRE lifecycle

Phase	AI-supported task	Required human checkpoint
Intake	Drafting project charter sections from initial request notes.	Project sponsor and Technical Lead confirm scope, approvals, and risk profile.
Needs Analysis	Competency gap mapping; learner profiling; first-draft learning objectives.	Clinical SME validates relevance, accuracy, and alignment to the competency framework.
Structure	Outline generation; storyboard drafting; branching-scenario scaffolds.	Instructional designer reviews pedagogy; SME reviews clinical plausibility.
Production	Clinical scenario scripts; non-photoreal medical illustration; voice-over support; first-pass assessment items.	SME sign-off on clinical content; accessibility review; psychometric review of assessments.
Implementation	Configuration scripts; first-draft learner communications; competency-tracking setup.	Technical Lead validates deployment; pilot cohort feedback gates full release.
Review	Feedback clustering across large response volumes; pattern detection in analytics; standards-checklist pre-scan.	Design team interprets findings; SME confirms remediation; governance committee signs off.
End Closure	Drafting of lessons-learned report from project artifacts.	Team validates accuracy; institutional knowledge owner archives final assets.

Two cautions are worth stating directly. First, the documented time savings from AI-supported instructional design —ranging from 50 to 70% for general course development [11] to 70–80% for simulation scenario authoring [7] —reflect the production effort, not the total project effort. Review, governance, and stakeholder coordination do not compress at the same rate. Reporting only the production gain overstates the end-to-end gain. Second, AI hallucination and bias remain live risks. INSPIRE’s response is procedural rather than rhetorical: every AI-generated artifact has a named human checkpoint in the workflow before it is allowed to move forward [3].

## Discussion

INSPIRE adds three things to the instructional design literature. It articulates an agile-phased hybrid as a recognizable category in its own right, rather than as a private compromise between ADDIE and SAM. It treats AI use as a first-class element of the design framework with explicit phase-by-phase mapping. And it embeds regulatory and quality logic within the model rather than treating compliance as a post hoc concern [4, 7]. These three moves are connected. AI has changed the speed of production, while regulatory expectations have continued to demand structured accountability; a framework that ignores either will not survive contact with practice.

### Practical contribution

The three moves above are not academic. Teams already using ADDIE can adopt INSPIRE without abandoning the phase structure they teach and audit against; teams already using SAM will recognize the iterative behavior inside each phase. What is new is the combination and the explicit roles it names — technical participation from intake, human-in-the-loop AI use across every phase, and continuous quality assurance rather than terminal inspection. For institutions that produce healthcare e-learning at an industrial scale, that combination makes the framework portable across projects rather than reinvented for each project.

### Healthcare-specific contribution

Healthcare e-learning is distinct from generic digital learning in three ways that INSPIRE is designed around. The evidence base moves faster than fixed curricula can track; the audit burden is higher than in most settings; and the downstream consequence of weak learning is patient harm rather than commercial inconvenience. INSPIRE responds by treating clinical SME involvement, regulatory alignment, and continuous quality assurance as design defaults rather than as project-specific overlays.

INSPIRE was developed and refined at the Saudi Commission for Health Specialties (SCFHS), the national regulator for postgraduate medical education and continuing professional development in the Kingdom of Saudi Arabia. The SCFHS environment made three implementation conditions unusually visible, and each shaped the framework. First, SCFHS scaled its accredited CPD system tenfold in two decades, from 1,000 to 10,500 activities [1]. A trajectory of that kind cannot be sustained through sequential design workflows; it requires processes that produce, review, and revise activities in parallel, and it was in meeting that requirement that the phase-based hybrid took its current form. Second, the clinical workforce is internationally trained, so content must remain aligned with multiple standards concurrently, and bilingual Arabic–English delivery is a routine requirement. AI-supported drafting and translation have made parallel-language production tractable at institutional scale, and the same capability now extends to additional languages spoken across the workforce, opening a wider access route than manual translation workflows could sustain. Third, SCFHS enforces continuous quality documentation at the activity level, which is why continuous review is embedded in the framework rather than added at the end. Two enabling factors work in the same direction: a centralized regulatory infrastructure that can pilot and codify methodological reforms quickly, and national digital-transformation mandates that create institutional appetite for AI-integrated workflows. The conditions named here are illustrative, not exclusive — any regulator or training board operating at scale, under external accreditation, and across multiple languages will face variants of the same pressures, and the operational features of the SCFHS setting are what an adopting institution should assess before implementing the framework.

## Conclusion

Healthcare e-learning is being asked to do more, faster, and to a higher standard, all at once. The instructional design models the field has relied on were not designed for that combination, and informal adaptation has only partially closed the gap. INSPIRE is offered here as a methodology built deliberately for this setting: a seven-phase agile-phased hybrid that pairs structured governance with iterative production, integrates technical roles from the start, places quality as a continuous function rather than a terminal one, and treats AI as part of the workflow rather than as a tool used outside it. The contribution is conceptual at this point, and it is presented as such. Empirical validation against time-to-delivery, learner outcomes, and audit-quality measures is the next stage of work, and a research agenda for that validation has been set out above. What we have tried to show in the meantime is that the gap INSPIRE addresses is real, that it is not closed by any single existing model, and that the framework offered here is coherent enough to be tested, applied, and revised in the open. Among the empirical questions that follow, one deserves particular emphasis: how INSPIRE compares against traditional instructional design models that have been actively augmented with AI capabilities by the teams already familiar with them. Distinguishing what INSPIRE contributes as a native design commitment from what any framework can accomplish through determined AI integration is a question that only comparative implementation research can answer.

## Data Availability

Data sharing is not applicable to this article, as no datasets were generated or analysed during the current study. All sources referenced in this article are publicly available through the citations provided.
